# Supplementary material for: Trefoil factors share a lectin activity that defines their role in mucus
Source: Nat Commun. 2020 May 13;11:2265. doi: 10.1038/s41467-020-16223-7 (PMC7221086; doi:10.1038/s41467-020-16223-7)
Supplement: Supplementary file 1 — Supplementary Information [file 41467_2020_16223_MOESM1_ESM.pdf]

<u>SUPPLEMENTARY INFORMATION</u>

Trefoil factors share a lectin activity that defines their role in mucus

Järvå, M. A. *et al.*

Supplementary Tables

Supplementary Table 1: Protein constructs used in this study. Mutations are in red, biotinylated Lys in green and Cys forming interchain disulfides in yellow.

construct	protein sequence
mTFF1	QTETCTVAPRERQNCGFPGVTPSQCANKGCCFDDTVRGVPWCFYPNTILEK
$mTFF1_{bio}$	MDHHHHHHGLNDIFEAQKIEWHEIDGRQTETCTVAPRERQNCGFPGVTPSQCANKGCCF DDTVRGVPWCFYPNTIDVPPEEECEF
$dTFF1_{bio}$	MDHHHHHHGLNDIFEAQKIEWHEIDGRQTETCTVAPRERQNCGFPGVTPSQCANKGCCF DDTVRGVPWCFYPNTIDVPPEEECEF
$dTFF1_{bio}$-N14A	MDHHHHHHGLNDIFEAQKIEWHEIDGRQTETCTVAPRERQACGFPGVTPSQCANKGCCF DDTVRGVPWCFYPNTIDVPPEEECEF
$dTFF1_{bio}$-W41A	MDHHHHHHGLNDIFEAQKIEWHEIDGRQTETCTVAPRERQNCGFPGVTPSQCANKGCCF DDTVRGVPACFYPNTIDVPPEEECEF
TFF2	EKPSPCQCSRLSPHQRTNCGFPGITSDQCFDNGCCFDSSVTGVPWCFHPLPKQESDQCV MEVSDRRNCGYPGISPEECASRKCCFSNFIFEVPWCFFPKSVEDCHYKHHHHHHHHHH
mTFF3	HHHHHHGLNDIFEAQKIEWHEIDGRGSEEYVGLSANQCAVPAKDRVDCGYPHVTPKECN NRGCCFDSRIPGVPWCFKPLQEAEST
$mTFF3_{bio}$	MDHHHHHHGLNDIFEAQKIEWHEIDGREEYVGLSANQCAVPAKDRVDCGYPHVTPKECN NRGCCFDSRIPGVPWCFKPLQEAECTF
$dTFF3_{bio}$	MDHHHHHHGLNDIFEAQKIEWHEIDGREEYVGLSANQCAVPAKDRVDCGYPHVTPKECN NRGCCFDSRIPGVPWCFKPLQEAECTF
$dTFF3_{bio}$-D20A	MDHHHHHHGLNDIFEAQKIEWHEIDGREEYVGLSANQCAVPAKDRVACGYPHVTPKECN NRGCCFDSRIPGVPWCFKPLQEAECTF
$dTFF3_{bio}$-W47A	MDHHHHHHGLNDIFEAQKIEWHEIDGREEYVGLSANQCAVPAKDRVDCGYPHVTPKECN NRGCCFDSRIPGVPACFKPLQEAECTF

Supplementary Table 2: Parameters used for ITC and thermodynamic terms determined for GlcNAc-α-1,4-Gal binding to the TFFs. Data are presented as mean values ±SD for three independent replicates.

**Protein (30 µM)**	**GlcNAc-α-1,4-Gal (µM)**	**T (°C)**	**n**	**K_d (µM)**	**ΔH (kcal · mol^{-1})**	**ΔS (cal · mol^{-1} · K^{-1})**	**-TΔS (kcal · mol^{-1})**	**ΔG (kcal · mol^{-1})**
mTFF1	1250	25	1.0 ± 0.2	49 ± 4	-10 ± 2	-14 ± 5	4 ± 2	-5.97 ± 0.03
TFF2	1250	25	0.7 ± 0.3	44 ± 8	-20 ± 10	-60 ± 40	20 ± 10	-6.2 ± 0.2
mTFF3	1250	25	1.0 ± 0.1	65 ± 5	-10.8 ± 0.5	-17 ± 2	5.0 ± 0.5	-5.83 ± 0.05

Supplementary Table 3: Refinement statistics for the structures reported in this study.

	**mTFF1 (PDB ID: 6V1D)**	**mTFF3:GlcNAc-α-1,4-Gal (PDB ID: 6V1C)**
**Data collection**		
Space group	P 1 2_1 1	P 4_1 2_1 2
chains per AU	3	1
Cell dimensions		
a, *b*, *c* (Å)	44.93, 41.90, 45.83	37.59, 37.59, 87.48
α, β, γ (°)	90, 115.57, 90	90, 90, 90
Wavelength (Å)	0.9537	0.9537
Resolution (Å)*	41.34-2.40 (2.49-2.40)	43.74-1.55 (1.58-1.55)
R_{sym} or R_{merge}*	0.231 (0.782)	0.081 (1.949)
R_{pim}*	0.225 (0.759)	0.035 (0.849)
$I / \sigma I$*	2.7 (1.0)	15.8 (1.3)
CC(1/2)*	0.958 (0.570)	0.999 (0.526)
Completeness (%)*	98.6 (97.3)	100 (100)
Redundancy*	3.3 (3.4)	11.5 (11.5)
Wilson B-factor (Å^2)	8.2	20.3
**Refinement**		
Resolution (Å)	41.34-2.40	34.54-1.55
No. reflections	6,046	9,706
R_{work} / R_{free}	0.1939 / 0.2259	0.1738 / 0.1882
No. non-H atoms		
Protein	1,114	404
Ligand/ion	n.a	26
Water	96	38
B-factors		
Protein	19.7	27.0
GlcNAc-α-1,4-Gal	n.a.	26.4
Water	21.5	37.6
R.m.s. deviations		
Bond lengths (Å)	0.002	0.009
Bond angles (°)	0.604	1.155
Ramachandran plot (%)		
Favored	97.81	97.62
Allowed	2.19	2.38
Disallowed	0	0

*Values in parentheses are for highest-resolution shell.

Supplementary Table 4: Synthetic dsDNA used to prepare expression plasmids in this study. Restriction sites are underlined.

sequence name	nucleotide sequence
mTFF1	AAAAACATATGAAATACCTGCTGCCGACCGCTGCTGCTGGTCTGCTGCTCCTCGCTGCCCAGCCGGCGATGGCC<br>CAGACCGAAACATGTACCGTAGCACCTAGAGAACGCCAGAACTGCGGCTTCCCGGGCGTGACGCCGTCACAATG<br>TGCTAATAAAGGCTGCTGTTTTGATGATACCGTTAGAGGCGTACCGTGGTGCTTCTATCCGAACACCATTCTCG<br>AGAAAAA
aviTFF1	AAAAACCATGGATCACCACCACCACCACCACGGTCTGAACGACATCTTCGAAGCGCAGAAGATCGAATGGCACG<br>AGATTGATGGCCGTCAAACCGAAACCTGCACCGTGGCGCCGCGTGAGCGTCAGAACTGCGGTTTTCCGGGCGTT<br>ACCCCGAGCCAATGCGCGAACAAAGGTTGCTGCTTCGACGATACCGTGCGTGGCGTTCCGTGGTGCTTTTACCC<br>GAACACCATTGACGTGCCGCCGGAGGAAGAGTGCGAGTTCTAACTCGAGAAAAA
aviTFF1-N14A	AAAAACCATGGATCACCACCACCACCACCACGGTCTGAACGACATCTTCGAAGCGCAGAAGATCGAATGGCACG<br>AGATTGATGGCCGTCAAACCGAAACCTGCACCGTGGCGCCGCGTGAGCGTCAGGCCTGCGGTTTTCCGGGCGTT<br>ACCCCGAGCCAATGCGCGAACAAAGGTTGCTGCTTCGACGATACCGTGCGTGGCGTTCCGTGGTGCTTTTACCC<br>GAACACCATTGACGTGCCGCCGGAGGAAGAGTGCGAGTTCTAACTCGAGAAAAA
$dTFF1_{bio}$-W41A	AAAAACCATGGATCACCACCACCACCACCACGGTCTGAACGACATCTTCGAAGCGCAGAAGATCGAATGGCACG<br>AGATTGATGGCCGTCAAACCGAAACCTGCACCGTGGCGCCGCGTGAGCGTCAGAACTGCGGTTTTCCGGGCGTT<br>ACCCCGAGCCAATGCGCGAACAAAGGTTGCTGCTTCGACGATACCGTGCGTGGCGTTCCGGCGTGCTTTTACCC<br>GAACACCATTGACGTGCCGCCGGAGGAAGAGTGCGAGTTCTAACTCGAGAAAAA
TFF2	AAAAAACTAGTAAATCAGTCACACCAAGGCTTCAATAAGGAACACACAAGCAAGATGGTAAGCGCTATTGTTTT<br>ATATGTGCTTTTGGCGGCGGCGGCGCATTCTGCCTTTGCGGAAAAGCCTTCTCCTTGTCAATGCTCTCGTCTGT<br>CTCCTCACCAACGTACCAACTGCGGTTTCCCTGGTATCACCTCTGATCAATGCTTCGACAACGGTTGCTGCTTC<br>GACTCTTCTGTGACCGGTGTGCCATGGTGCTTCCACCCTCTGCCTAAGCAAGAATCTGACCAATGCGTGATGGA<br>AGTGTCTGACCGTCGTAACTGCGGTTACCCTGGTATCTCTCCTGAAGAATGCGCTTCTCGTAAGTGCTGCTTCT<br>CTAACTTCATCTTCGAAGTGCCTTGGTGCTTCTTCCCTAAGTCTGTGGAAGACTGCCACTACAAACACCATCAT<br>CACCACCATCACCACCATCACTGACTCGAGAAAAA
mTFF3	AAAAACATATGAAATACCTGCTGCCGACCGCTGCTGCTGGTCTGCTGCTCCTCGCTGCCCAGCCGGCGATGGCC<br>GAAGAATATGTAGGACTATCAGCTAACCAGTGTGCGGTACCGGCGAAAGATCGCGTTGATTGCGGTTATCCGCA<br>TGTGACGCCGAAAGAATGTAACAACCGCGGCTGCTGCTTTGATAGCCGTATTCCGGGCGTGCCGTGGTGTTTTA<br>AACCGCTGCAGGAAGCGGAAAGCACCCTCGAGAAAAA
aviTFF3	AAAAACCATGGATCACCACCACCACCACCACGGTCTGAACGACATCTTCGAGGCGCAGAAGATCGAGTGGCACG<br>AAATTGATGGTCGTGAGGAATACGTTGGTCTGAGCGCGAACCAATGCGCGGTGCCGGCGAAGGACCGTGTTGAT<br>TGCGGCTATCCGCACGTGACCCCGAAAGAATGCAACAACCGTGGTTGCTGCTTTGACAGCCGTATTCCGGGCGT<br>TCCGTGGTGCTTCAAACCGCTGCAGGAAGCGGAATGCACCTTTTAACTCGAGAAAAA
aviTFF3-D20A	AAAAACCATGGATCACCACCACCACCACCACGGTCTGAACGACATCTTCGAGGCGCAGAAGATCGAGTGGCACG<br>AAATTGATGGTCGTGAGGAATACGTTGGTCTGAGCGCGAACCAATGCGCGGTGCCGGCGAAGGACCGTGTTGCT<br>TGCGGCTATCCGCACGTGACCCCGAAAGAATGCAACAACCGTGGTTGCTGCTTTGACAGCCGTATTCCGGGCGT<br>TCCGTGGTGCTTCAAACCGCTGCAGGAAGCGGAATGCACCTTTTAACTCGAGAAAAA
aviTFF3-W47A	AAAAACCATGGATCACCACCACCACCACCACGGTCTGAACGACATCTTCGAGGCGCAGAAGATCGAGTGGCACG<br>AAATTGATGGTCGTGAGGAATACGTTGGTCTGAGCGCGAACCAATGCGCGGTGCCGGCGAAGGACCGTGTTGAT<br>TGCGGCTATCCGCACGTGACCCCGAAAGAATGCAACAACCGTGGTTGCTGCTTTGACAGCCGTATTCCGGGCGT<br>TCCGGCGTGCTTCAAACCGCTGCAGGAAGCGGAATGCACCTTTTAACTCGAGAAAAA

Supplementary Figures

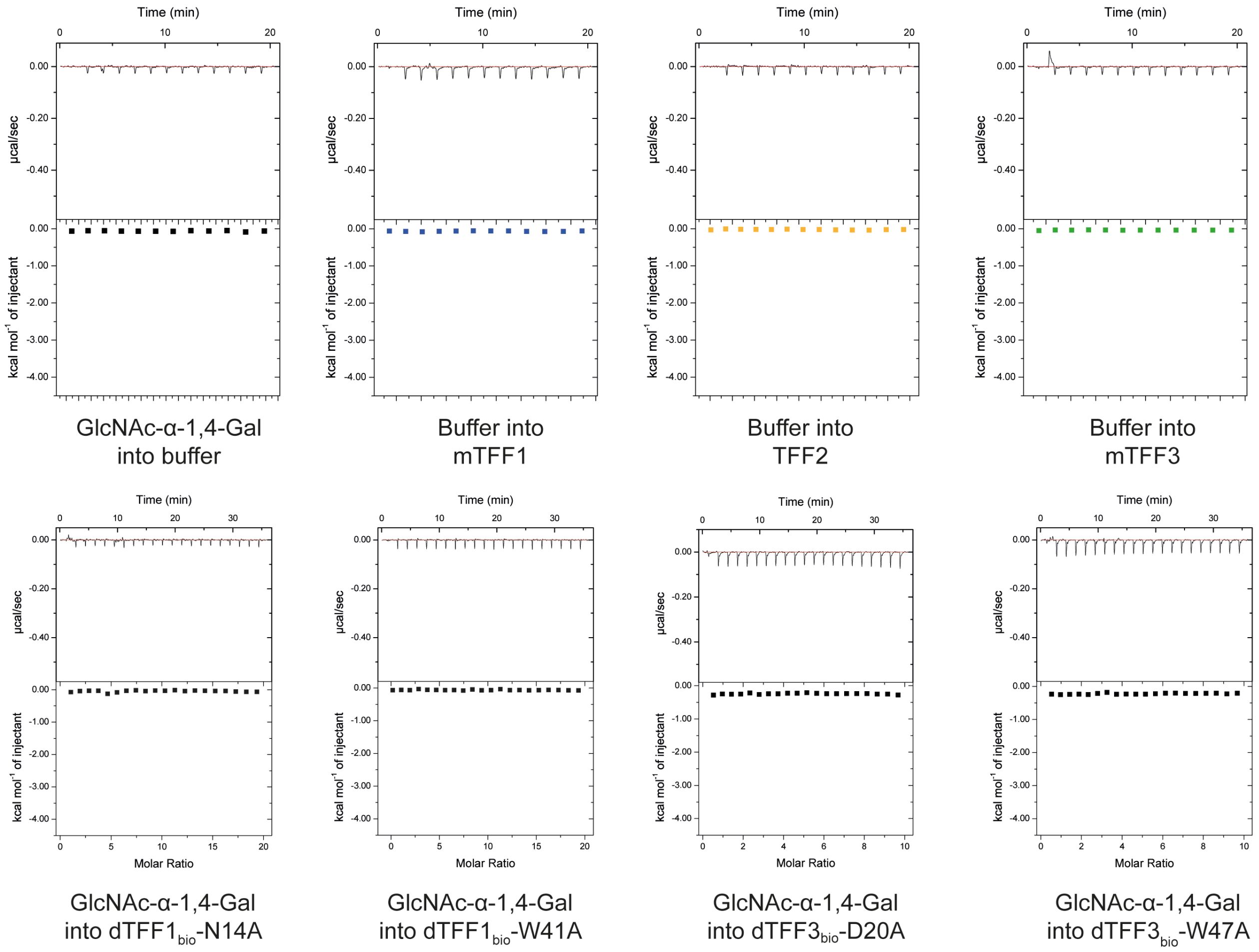

Supplementary Figure 1: ITC controls and negative results. Top row: Ligand into buffer and buffer into protein controls for Figure 1D. Bottom row: ITC data for TFF mutants.

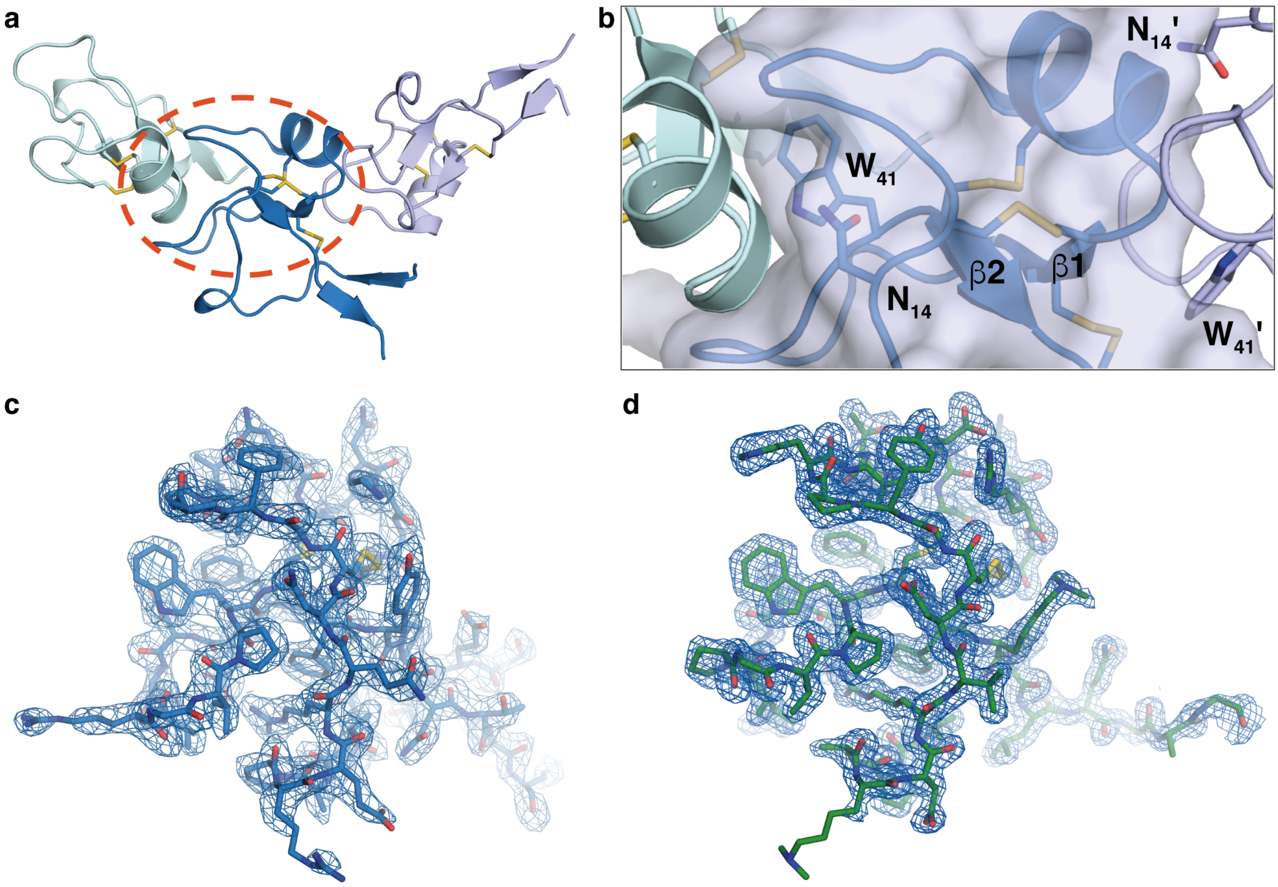

Supplementary Figure 2: The crystal structures of human TFF1 and TFF3. The apo mTFF1 crystal structure has (a) three molecules in the asymmetric unit and (b) crystallographic contacts that occupy the ligand-binding site. (c) The structure of mTFF1 with a 2Fo-Fc map contoured at 1.0σ. (d) The structure of mTFF3 (ligand removed) with a 2Fo-Fc map contoured at 1.0σ.

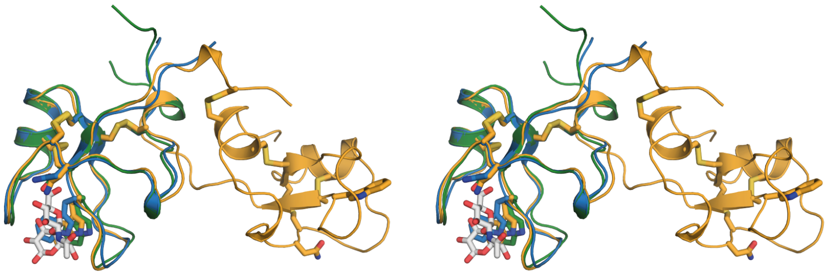

Supplementary Figure 3: A superposition of the TFFs in stereoview. A superposition of TFF3–GlcNAc-α-1,4-Gal (green), TFF1 (blue) and porcine TFF2 (orange) (PDB ID: 2PSP) in stereoview.

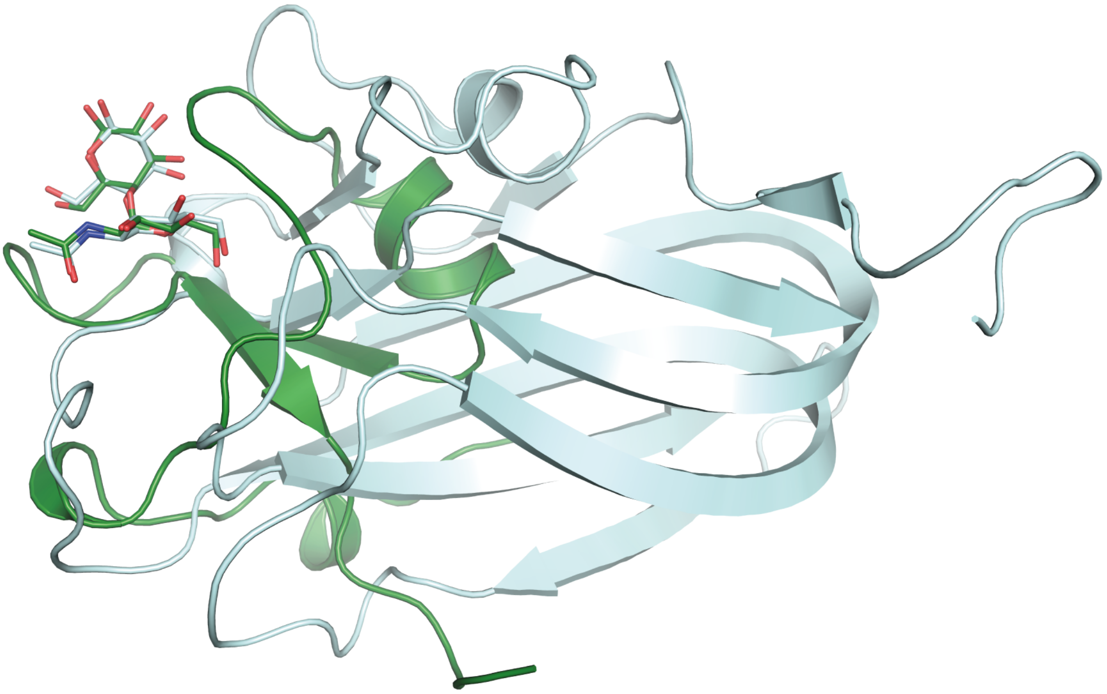

Supplementary Figure 4: CBM32–TFF3 superposition. A superposition of a CBM32 domain (PDB ID: 4A6O, cyan) and mTFF3 (green) aligned through their GlcNAc-α-1,4-Gal ligand.

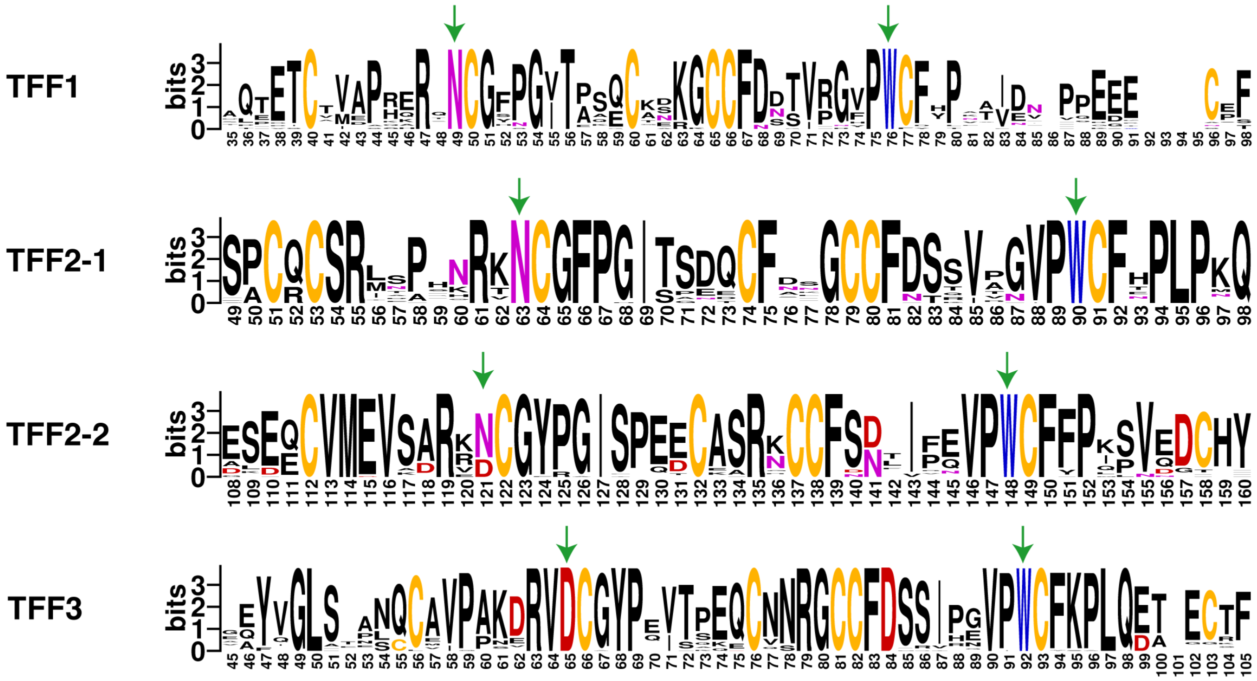

Supplementary Figure 5: Web-logos illustrating sequence conservation in the trefoil domains of mammalian TFF1-3. The residues with sidechains that interact with the GlcNAc-α-1,4-Gal ligand are indicated with green arrows.

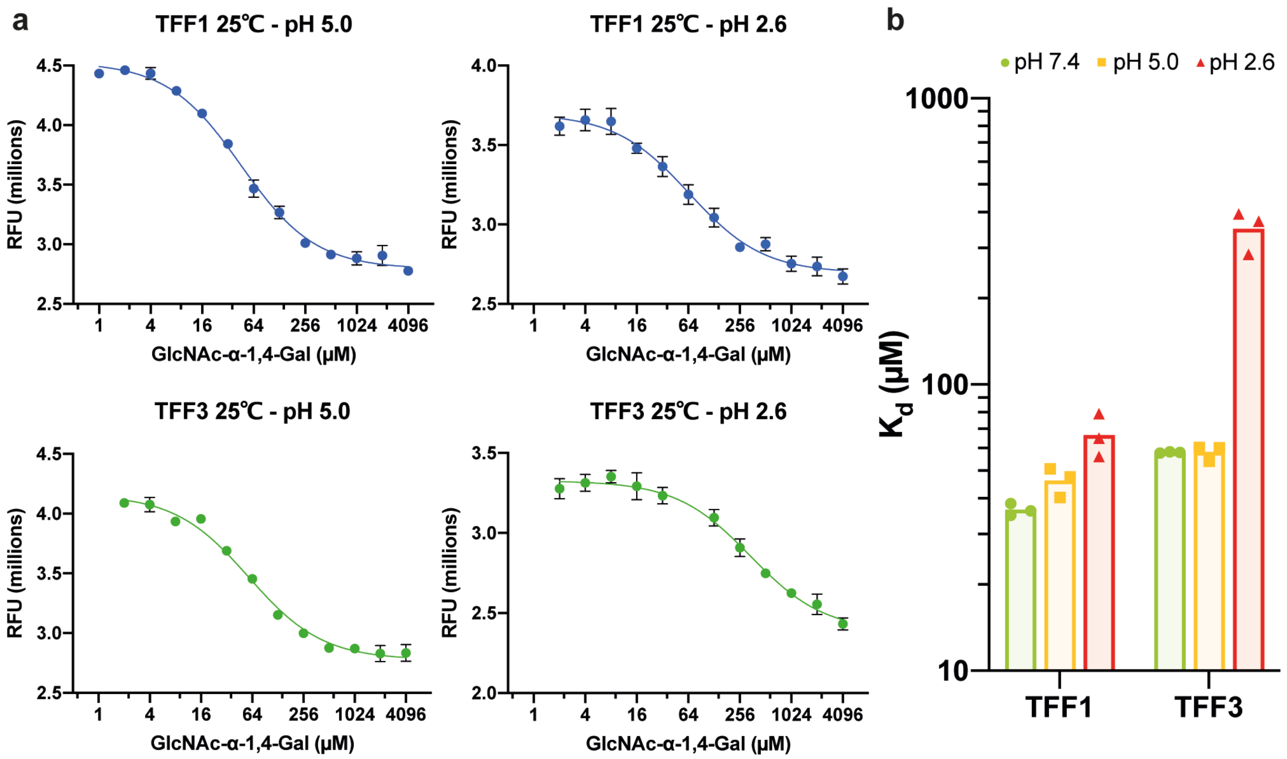

Supplementary Figure 6: Tryptophan fluorescence quenching assays at different pH for GlcNAc-α-1,4-Gal binding to mTFF1 and mTFF3. (a) The titration curves for pH 5.0 and for pH 2.6 with the one-site curve fit. Data are presented as mean values ±SD for three independent replicates. (b) Bar-graph of the average and individual log(K_d) for each pH and TFF. Source data are provided as a Source Data file.

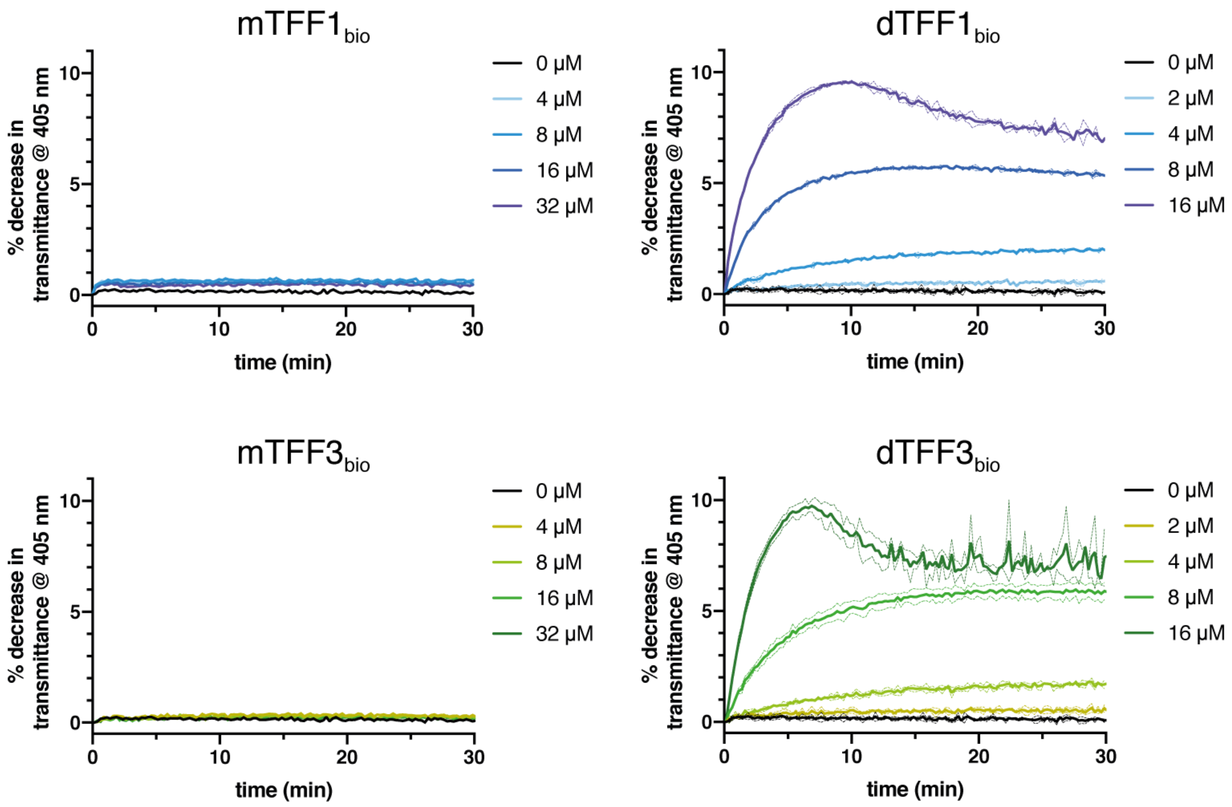

Supplementary Figure 7: Agglutination of pMucin at different concentrations of monomeric and dimeric TFF1 and TFF3. Curves are averaged from three replicates and dotted lines indicate standard deviations. Source data are provided as a Source Data file.

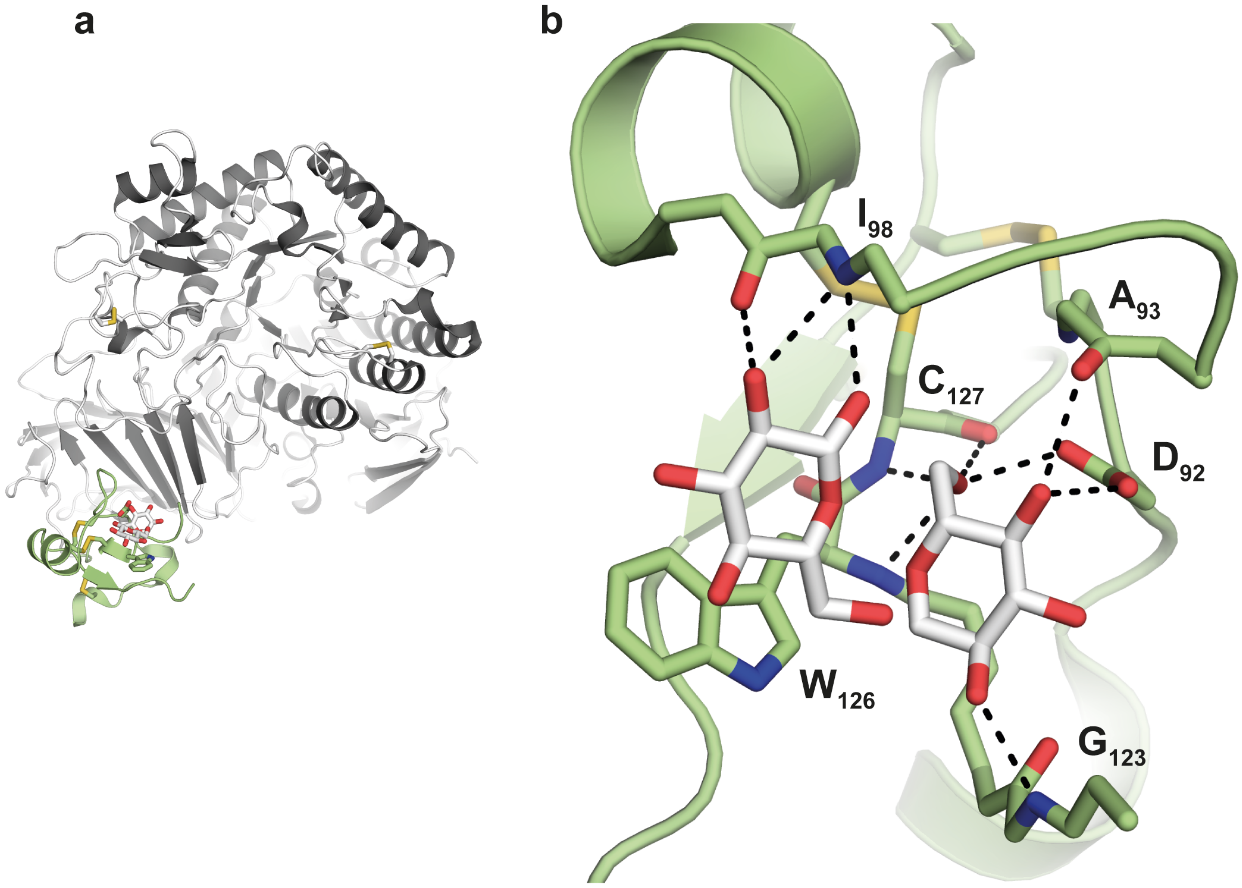

Supplementary Figure 8: the ligand bound to the trefoil domain of human lysosomal α-glucosidase. (a) The N-terminal trefoil domain (green cartoon) in human lysosomal α-glucosidase (grey cartoon) (PDB ID: 5KZW) is bound to isomaltose (white sticks with oxygen atoms coloured red). (b) Close-up of the trefoil isomaltose binding site with residues forming hydrogen bonds drawn in sticks (sulfur, nitrogen and oxygen coloured yellow, blue and red, respectively).

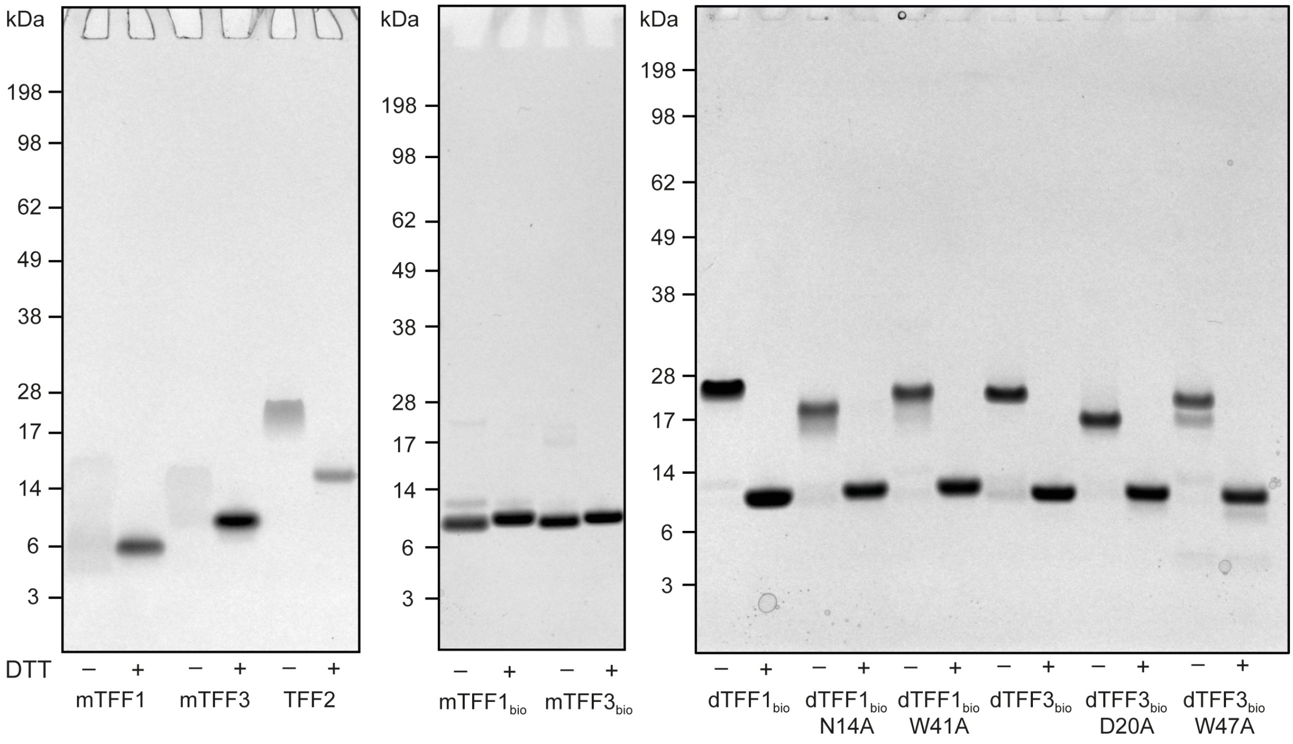

Supplementary Figure 9: SDS PAGE gels of TFF samples. Non-reducing and reducing SDS-PAGE gels of monomeric TFF1/3 and TFF2 (mTFF1, mTFF3, TFF2); biotinylated monomeric TFF1/3 ($mTFF1_{bio}$, $mTFF3_{bio}$); and biotinylated dimeric TFF1/3 ($dTFF1_{bio}$ and $dTFF3_{bio}$) and mutants thereof. Protein purity is representative of that obtained for two or more independently produced samples. Source data are provided as a Source Data file.

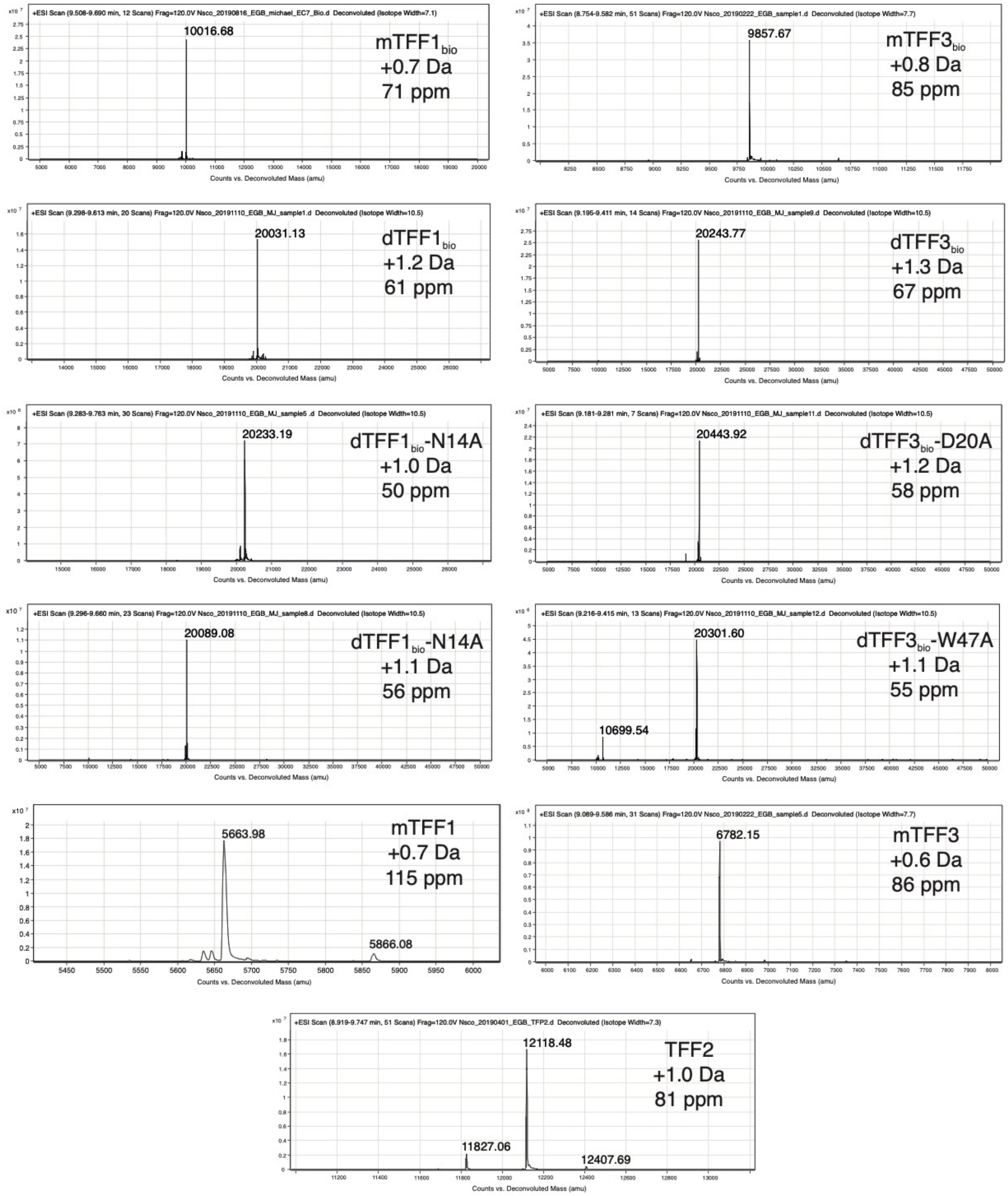

Supplementary Figure 10: Deconvoluted ESI-MS mass spectra of recombinant TFFs. The mass $[M]^+$ of reverse phase separated TFF samples are ±1.5 Da of the expected masses.
